# Assessment of Traffic Accidents in Japan during the COVID-19 Pandemic vs. Previous Years: A Preliminary Report

**DOI:** 10.3390/healthcare10050860

**Published:** 2022-05-06

**Authors:** Kazuki Shimizu, Peter Ueda, Cyrus Ghaznavi, Haruka Sakamoto, Shuhei Nomura

**Affiliations:** 1 Department of Health Policy, London School of Economics and Political Science, London WC2A 2AE, UK; 2 Faculty of Public Health and Policy, London School of Hygiene and Tropical Medicine, London WC1E 7HT, UK; 3 Tokyo Foundation for Policy Research, Tokyo 106-6234, Japan; harukask@m.u-tokyo.ac.jp (H.S.); s-nomura@m.u-tokyo.ac.jp (S.N.); 4 Department of Global Health Policy, Graduate School of Medicine, The University of Tokyo, Tokyo 113-0033, Japan; peter.ueda@gmail.com; 5 Department of Health Policy and Management, School of Medicine, Keio University, Tokyo 160-8582, Japan; cghaznavi@gmail.com; 6 Medical Education Program, Washington University School of Medicine, St. Louis, MI 63110-1010, USA; 7 Department of International Affairs and Tropical Medicine, Tokyo Women’s Medical University, Tokyo 162-8666, Japan

**Keywords:** COVID-19, Japan, public health and social measures, traffic accident, monitoring, evaluation

## Abstract

Beginning in April 2020, social distancing measures were implemented to mitigate the COVID-19 pandemic in Japan. We assessed whether traffic accident rates had decreased from April 2020 to December 2021 as compared with previous years. The analysis included 2,934,477 traffic accidents, and the trend of decreasing rates of traffic accidents in recent years and seasonal fluctuations in traffic accidents were considered. The yearly change in the traffic accident rate between 2015 and 2019 was estimated, and the traffic accident rate in 2020 and 2021 was predicted. This was followed by the comparison of observed vs. predicted traffic accident rate. In 2020, the observed vs. expected rates of traffic accidents were lower in April to December 2020, and the rate of traffic accidents in Japan was 30–40% lower in April–May 2020 than would be expected based on trends from previous years. In 2021, rates of traffic accidents remained lower than expected between January and November, but the magnitude of decrease was not as pronounced. These findings could be explained by social distancing policies, including the declaration of the state of emergency, and the relaxation of public health and social measures over time.

## 1. Introduction

The Coronavirus Disease 2019 (COVID-19) pandemic led many countries to impose public health and social measures (PHSMs) aimed at decreasing and suppressing transmission. These included unprecedented social distancing policies such as lockdown and curfews. Due to COVID-19, health system capacity was overstretched in many countries [1], and this negatively impacted the maintenance of other essential health services. However, some positive outcomes in public health have been reported during the pandemic. In particular, downward trends in the number of injuries and deaths associated with accidents have been reported in many countries [2,3,4,5,6], including decreased morbidity and mortality due to road injuries.

In Japan, the first state of emergency was declared in April–May 2020 to suppress a surge of COVID-19 cases. During this period, citizens were requested to refrain from going out except for activities necessary for maintaining their lives and livelihoods. While no mandatory measures regarding transportation and travel, such as night-time curfews or domestic travel restrictions, were implemented, entertainment and commercial facilities were requested to suspend their business and schools were closed [7,8]. The decrease in mobility and traffic volumes during this period has been confirmed by multiple sources [9,10]. For example, traffic volumes on the expressway in the entirety of Japan compared with the previous year had decreased by over 25% during 5–11 April 2020, which was followed by a significant decrease of 70% during Japan’s holiday season from 25 April to 6 May 2020 [10]. Previous research has also clarified that the number of deaths due to road injuries had marginally decreased in the several weeks during the state of emergency [11]. In addition, due to the resurgence of COVID-19, the second state of emergency was declared for the Greater Tokyo Area and other prefectures where the magnitude of COVID-19 was judged as relatively severe for maintaining health system capacity in January–March 2021 and January–February 2021, respectively [12]. Despite these, the poor control of COVID-19 continued [13], which necessitated the third state of emergency in Tokyo and other metropolitan areas in April–June 2021, whereas the quasi-state of emergency was also declared to many municipalities during the same period [14,15]. Furthermore, to cope with the resurgence of COVID-19, the fourth state of emergency was declared to several prefectures including Tokyo where the Olympic and Paralympic Games were held without spectators [15,16].

While Japan has recorded a continued decline in the number of road injuries for over a decade along with a consistent decrease in fatalities through comprehensive interventions, including traffic regulations and law enforcement, education to the public, improved performance of vehicle safety standards, and high-standard emergency medical care [17], traffic accidents, regardless of their severity, have an impact on healthcare provision, especially in emergency departments. Therefore, continued evaluation of trends in the total incidence of road injuries is important. This also helps assess the impact of PHSMs aimed to curb the pandemic. However, little is known about the incidence of road injuries in Japan in the context of COVID-19. In this study, we assessed monthly traffic accident rates in 2015–2021 and examined whether rates of road injuries in 2020–2021 differed from expected rates based on trends from previous years.

## 2. Materials and Methods

In Japan, the National Police Agency publicly compiles national data on the number of traffic accidents that involve fatal or non-fatal injuries of people occurring on the road, as stipulated by the Road Traffic Act [18]. We obtained data on the monthly number of traffic accidents in Japan between January 2015 to December 2021 from the National Police Agency website [19]. We used an analytical approach [20] that accounted for the trend of decreasing rates of traffic accidents in recent years and for the seasonal fluctuations in traffic accidents. First, we calculated the rate of traffic accidents for each month by dividing the number of traffic accidents by the total population. For each month separately, we then estimated the yearly change in the traffic accident rate between 2015 and 2019 by using linear regression models with the logarithm of the traffic accident rate as the dependent variable and year as a continuous independent variable as follows:

ln (rate) = α_i_ + β_i_ × year, (1)

where i represents month and rate represents the traffic accident rate per 100,000 population.

We then used these models to predict the traffic accident rate in 2020 and 2021 as follows and calculated the ratio of observed vs. predicted traffic accident rate.

ln (rate) _2020, 2021_ = α_i_ + β_i_ × year _(2020, 2021)_, (2)

In total, 95% confidence intervals (CIs) for the ratios were calculated based on standard errors calculated using the number of traffic accidents. The difference between the observed and predicted rate was considered statistically significant if the 95% CI of the ratio did not overlap 1. We used Stata version 16.1 (StataCorp). Institutional board review was not required because no individual-level data were used.

## 3. Results

**Figure 1:**
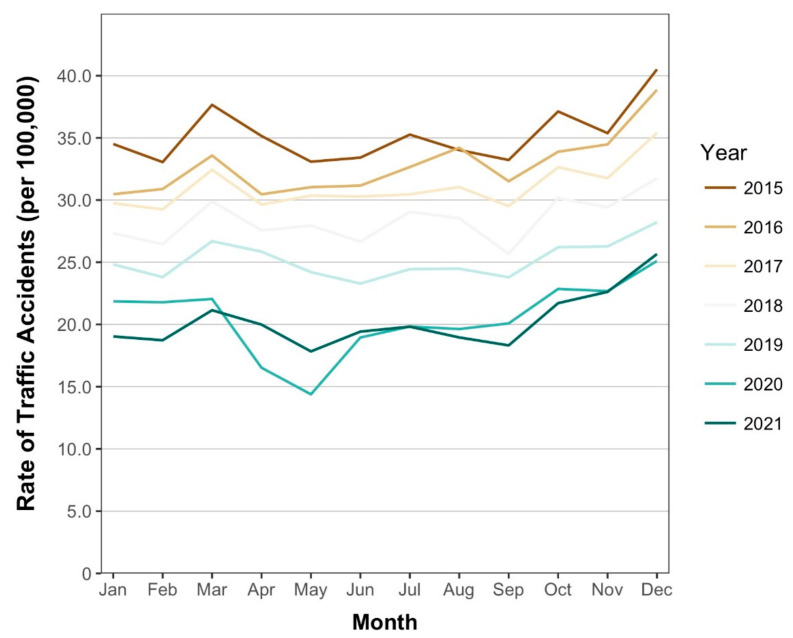
Monthly rates of traffic accidents in Japan, 2015–2021.

Analyses were based on 2,934,477 traffic accidents from January 2015 to December 2021. Monthly rates of traffic accidents for each year are shown in Figure 1.

**Figure 2:**
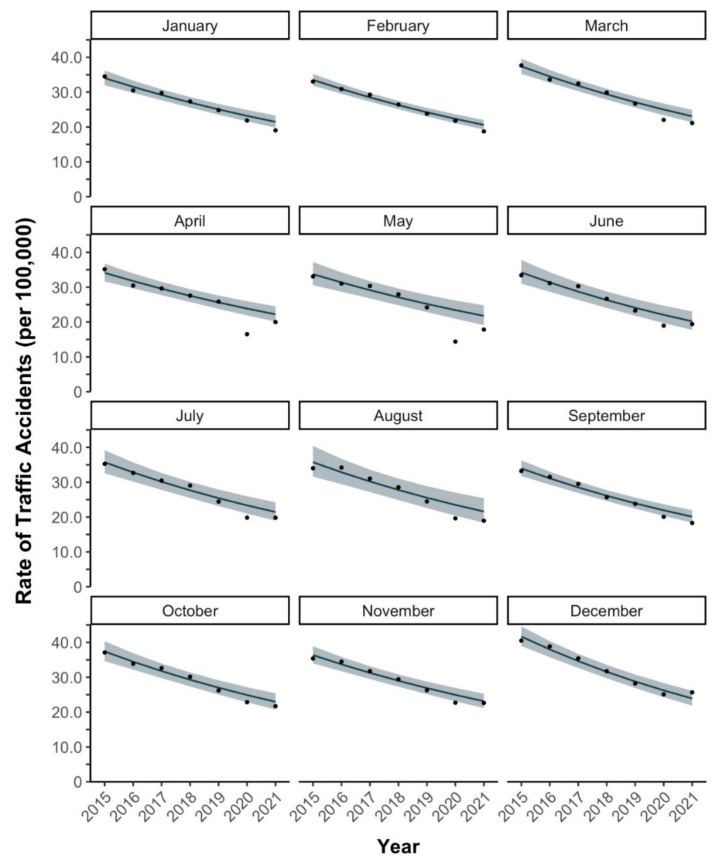
Monthly observed vs. expected rates of traffic accidents in Japan in 2015–2021. Dots represent observed rates, lines represent expected rates, and shaded areas represent 95% CI for expected rates.

Between 2015 and 2019, rates of traffic accidents generally decreased in investigated months, with the yearly decrease ranging from 7.2 to 9.3% (Table 1, Figure 2). In 2020, the number of traffic accidents (rate per 100,000 population) was 27,523 (21.8) in January, 27,443 (21.8) in February, 27,763 (22.0) in March, followed by a dip to 20,805 (16.5) in April and 18,107 (14.4) in May. The number marginally increased to 23,846 (18.9) in June, followed by a continuous but gradual increase to 31,551 (25.1) in December. In 2021, the number of traffic accidents decreased to 23,896 (19.0) in January. A marginal increase to 26,523 (21.1) in March was followed by a dip to 22,373 (17.8) in May. Since September 2021, the number continued to gradually increase, and amounted to 32,196 (25.7) in December.

**Figure 3:**
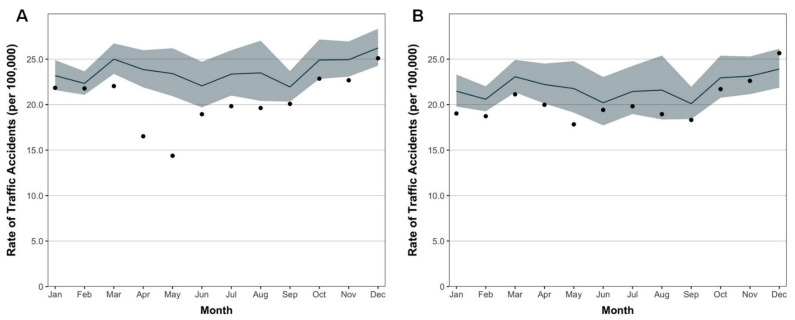
Monthly observed vs. expected rates of traffic accidents in Japan. (**A**) The year 2020. (**B**) The year 2021. Dots represent observed rates, lines represent expected rates, and shaded areas represent 95% CI for expected rates.

Figure 3A,B presents a comparison of observed and expected rates of traffic accidents by month in 2020 and 2021, respectively. In 2020, the observed rates of traffic accidents in 2020 were lower than expected rates in all months, with large differences observed in April (16.5 vs. 23.9 traffic accidents per 100,000 population; RR, 0.69 [95%CI, 0.68–0.70]) and May (14.4 vs. 23.4 traffic accident per 100,000 population; RR, 0.61 [95%CI, 0.61–0.62]). The observed rates of traffic accidents in 2020 remained lower than expected rates during the remaining months of the year, although the differences were not as pronounced as in April and May: June (18.9 vs. 22.1 traffic accidents per 100,000 population; RR, 0.86 [95%CI, 0.85–0.87]), July (19.8 vs. 23.4 traffic accidents per 100,000 population; RR, 0.85 [95%CI, 0.84–0.86]), August (19.6 vs. 23.5 traffic accidents per 100,000 population; RR, 0.84 [95%CI, 0.83–0.85]), September (20.1 vs. 21.9 traffic accidents per 100,000 population; RR, 0.92 [95%CI, 0.90–0.93]), October (22.9 vs. 24.9 traffic accidents per 100,000 population; RR, 0.92 [95%CI, 0.91–0.93]), November (22.7 vs. 24.9 traffic accidents per 100,000 population; RR, 0.91 [95%CI, 0.90–0.92]) and December (25.1 vs. 26.2 traffic accidents per 100,000 population; RR, 0.96 [95%CI, 0.95–0.97]). In 2021, the observed rates of traffic accidents were lower than expected rates in January-November, with the large differences observed in May (17.8 vs. 21.8 traffic accidents per 100,000 population; RR, 0.82 [95%CI, 0.81–0.83]) and August (19.0 vs. 21.6 traffic accident per 100,000 population; RR, 0.88 [95%CI, 0.87–0.89]). The observed rates of traffic accidents in 2021 remained lower than expected rates by November 2021, although the differences were not as pronounced as in May and August: September (18.3 vs. 20.1 traffic accidents per 100,000 population; RR, 0.91 [95%CI, 0.90–0.92]), October (21.7 vs. 23.0 traffic accidents per 100,000 population; RR, 0.95 [95%CI, 0.93–0.96]), and November (22.6 vs. 23.1 traffic accidents per 100,000 population; RR, 0.98 [95%CI, 0.97–0.99]). In December 2021, the observed rate of traffic accidents recorded higher than expected rates (25.7 vs. 23.9 traffic accidents per 100,000 population; RR, 1.07 [95%CI, 1.06–1.08]). The results of the analyses in detail are shown in Supplementary Table S1.

## 4. Discussion

We found that the rate of traffic accidents in Japan was 30–40% lower in April-May 2020 than would be expected based on trends from previous years. The rates of traffic accidents remained lower than expected during the remaining months of the year 2020, although the magnitude of decrease was not as pronounced. These findings might be explained by social distancing policies, including the declaration of the state of emergency for 7 prefectures on 7 April 2020 that expanded to the entire nation on 16 April 2020, which encouraged citizens to stay home and avoid environments where the likelihood of transmission is high [7,8]. Similar results have been shown in studies from other countries and regions that investigated the impact of lockdown on human mobility and traffic accidents [2,3,4,5,6]. Previous research on Japan has found that the number of fatalities due to road injuries had slightly decreased in early to mid-2020 compared with previous years [11]. Given Japan’s overstretched healthcare system, and a relatively limited intensive care unit capacity compared with other developed countries [21,22], the decrease in traffic accidents has concomitantly decreased the burden on emergency medical services and might have allowed for more resources to be devoted to treating COVID-19 patients. Moreover, although the state of emergency was lifted in 39 prefectures on 19 May 2020, followed by a full relaxation in the entirety of Japan in late May 2020, rates of traffic accidents were lower than expected also in June–December 2020. This might partially reflect Japanese residents’ efforts to adhere to social distancing policies that remained in place for the remainder of the year and other general recommendations for minimizing mobility, such as an increase in teleworking from home [23].

In 2021, the rates of traffic accidents remained lower than expected in most of the months, and the decreasing trend was particularly pronounced in January, May and August. These corresponded to the periods when the resurgence of COVID-19 occurred in Japan. However, it should be simultaneously argued that the magnitude of decrease in these months was not as pronounced as the year 2020. Less stringent countermeasures under the scope of state of emergency and quasi-state of emergency, and subsequent changes of behavior among citizens, could be considered as potential reasons. Interestingly, whereas the observed rates did not exceed expected rates, a gradual and continued increase in observed rates of traffic accidents was observed from September 2021; furthermore, in December 2021, observed rate exceeded the predicted rate, which was assumed to be partially brought by the relaxation of PHSMs and increased mobility for festive season, along with the successful suppression of COVID-19 in Japan.

It is encouraging that there has been a decreasing trend in the rates of traffic accidents in Japan during the past 6 years. This trend might be partly attributable to the measures aimed at reducing traffic accidents put in place through the Fundamental Traffic Safety Program [24] but could also be due to other factors such as changes in the sociodemographic composition of drivers and their behaviors, such as an increase in voluntary surrenders of driver’s licenses among the elderly who might suffer from cognitive impairment and/or a decreased car ownership ratio among the young generation aged below 30, which might yield a decrease in the absolute number of traffic accidents.

A limitation of our study is the lack of data on the type of traffic accident, its severity, and the gender and age of the driver. Although an increased proportion of deaths due to speed-related traffic accidents compared with other causes was reported in Japan in early 2020 [25], there has been a reporting lag for data on traffic accident in Japan that is freely accessible on the order of a few years; thus, data stratified by categories was not available. More detailed analyses are warranted, and this paper will be used as a launchpad. Moreover, there might have been differences between observed and expected rates that are not due to the pandemic, and it is not plausible to interpret that all findings in this study were attributed to the COVID-19 pandemic. Further research may also be needed to assess trends at the sub-national level. Despite these limitations, our analyses could address challenges in the official report published by the National Policy Agency that only compares the latest data with the previous year, expand on the previous knowledge by predicting the expected rates based on previous trends and comparing them with the observed rates during the COVID-19 pandemic by accounting for the declining trend in traffic accidents during the past 6 years preceding the COVID-19 pandemic, and provide assessment of the trend of traffic accidents that was interpretable from existing empirical data.

## 5. Conclusions

Based on trends from previous years, the rates of traffic accidents in Japan in April to December 2020 were lower than the expected rates, and this pattern was particularly pronounced during the state of emergency period in April–May 2020. The trend continued by November 2021, with the relatively pronounced pattern observed in January, May, and August 2021; however, in December 2021, the rate of traffic accidents in Japan became higher than the expected rate. More detailed analysis by category and further research at the sub-national level will bring in-depth assessment.

## Figures and Tables

**Table 1:** Yearly change of rates of traffic accidents in Japan by months.

Month	Yearly Change 2015–2019, % (95% CI)
January	−7.7 (−10.2 to −5.2)
February	−8.1 (−10.2 to −6.1)
March	−8.1 (−10.4 to −5.7)
April	−7.2 (−10.2 to −4.1)
May	−7.3 (−11.3 to −3.3)
June	−8.8 (−12.8 to −4.8)
July	−8.5 (−12.3 to −4.7)
August	−8.4 (−13.4 to −3.4)
September	−8.7 (−11.4 to −6.0)
October	−8.1 (−11.2 to −5.0)
November	−7.5 (−10.3 to −4.8)
December	−9.3 (−12.0 to −6.5)

## Data Availability

The data are publicly available at websites cited on references [19].

## Supplementary Materials

The following supporting information can be downloaded at: https://www.mdpi.com/article/10.3390/healthcare10050860/s1, Table S1: Monthly observed vs expected rates of traffic accidents in Japan in 2020–2021.
