# Supplementary material for: Assessment of Traffic Accidents in Japan during the COVID-19 Pandemic vs. Previous Years: A Preliminary Report
Source: Healthcare (Basel). 2022 May 6;10(5):860. doi: 10.3390/healthcare10050860 (PMC9141637; doi:10.3390/healthcare10050860)
Supplement: Supplementary file 1 [file healthcare-10-00860-s001.zip › healthcare-1675488-supplementary.pdf]

Supplementary Table S1. Monthly observed vs expected rates of traffic accidents in Japan in 2020–2021.

Year	Month	Observed Rate	Expected Rate (95% CI)	Observed vs. Expected Rate Ratio
2020	January	21.8	23.2 (21.6–24.9)	0.94 (0.93–0.95)
	February	21.8	22.3 (21.1–23.7)	0.98 (0.96–0.99)
	March	22.0	25.0 (23.4–26.7)	0.88 (0.87–0.89)
	April	16.5	23.9 (21.9–26.0)	0.69 (0.68–0.70)
	May	14.4	23.4 (20.9–26.2)	0.61 (0.61–0.62)
	June	18.9	22.1 (19.7–24.7)	0.86 (0.85–0.87)
	July	19.8	23.4 (21.0–26.0)	0.85 (0.84–0.86)
	August	19.6	23.5 (20.4–27.0)	0.84 (0.83–0.85)
	September	20.1	21.9 (20.3–23.7)	0.92 (0.90–0.93)
	October	22.9	24.9 (22.8–27.2)	0.92 (0.91–0.93)
	November	22.7	24.9 (23.1–26.9)	0.91 (0.90–0.92)
	December	25.1	26.2 (24.3–28.3)	0.96 (0.95–0.97)
2021	January	19.0	21.5 (19.8–23.3)	0.89 (0.87–0.90)
	February	18.7	20.6 (19.3–22.0)	0.91 (0.90–0.92)
	March	21.1	23.1 (21.4–24.9)	0.92 (0.91–0.93)
	April	20.0	22.2 (20.1–24.5)	0.90 (0.89–0.91)
	May	17.8	21.8 (19.1–24.8)	0.82 (0.81–0.83)
	June	19.4	20.2 (17.7–23.0)	0.96 (0.95–0.97)
	July	19.8	21.5 (19.0–24.3)	0.92 (0.91–0.94)
	August	19.0	21.6 (18.4–25.4)	0.88 (0.87–0.89)
	September	18.3	20.1 (18.4–22.0)	0.91 (0.90–0.92)
	October	21.7	23.0 (20.8–25.4)	0.95 (0.93–0.96)
	November	22.6	23.1 (21.2–25.3)	0.98 (0.97–0.99)
	December	25.7	23.9 (21.9–26.1)	1.07 (1.06–1.08)
